# The ability of phase angle and body composition to predict risk of death in maintenance hemodialysis patients

**DOI:** 10.1007/s11255-023-03708-9

**Published:** 2023-08-05

**Authors:** Yuanzhao Xu, Shuyi Ling, Zheyan Liu, Denggui Luo, Airong Qi, Youjia Zeng

**Affiliations:** Shenzhen Traditional Chinese Medicine Hospital, The Fourth Clinical Medical College of Guangzhou University of Chinese Medicine, Shenzhen, 518000 Guangdong China

**Keywords:** Maintenance hemodialysis, Risk of death, Phase angle, Hypoproteinemia

## Abstract

**Objective:**

The objective of this study was to investigate the ability of phase angle and body composition to identify risk factors for mortality among patients receiving maintenance hemodialysis (MHD) treatment.

**Methods:**

In this retrospective study, we examined the causes of death in 43 MHD patients who were treated at our hemodialysis center between January 2016 and December 2021 and compared the patients to 71 patients who survived during the same period. Body composition was measured using direct segmental multi-frequency bioelectrical impedance to obtain phase angle, fat-free mass (FFM), extracellular water/total body water (ECW/TBW), and waist circumference (WC). Laboratory data were also collected. Phase angle cut-off value-associated variables were identified using ROC analysis. The ability of body composition variables to identify risk factors for death in MHD patients was evaluated.

**Results:**

We found that cardiovascular disease was the most common cause of death among MHD patients. ROC curve analysis revealed that the optimal cut-off value for phase angle as a predictor of death risk in MHD patients was 4.50°. Additionally, lower phase angle, increased age, longer dialysis vintage, lower KT/V, and hypoproteinemia were identified as significant risk factors for death in MHD patients.

**Conclusion:**

In conclusion, our findings suggest that cardiovascular disease is the leading cause of death among MHD patients and that lower phase angle, increased age, longer dialysis duration, and hypoproteinemia can be used to predict the risk of mortality in this patient population. The underlying mechanism by which lower phase angle can be used to predict the prognosis of MHD patients warrants further investigation.

## Introduction

The incidence of chronic kidney disease (CKD) is increasing globally [1]. As the disease progresses, some CKD patients develop end-stage renal disease (ESRD), necessitating high medical resources for renal replacement therapy (RRT) and management of complications, resulting in a significant economic burden. Hemodialysis is a principal RRT strategy. Key goals for nephrologists are to reduce the mortality rate, improve long-term survival, reduce medical costs, improve the quality of life, and minimize economic burden for hemodialysis patients as much as possible [2]. Recent studies have explored bioelectrical impedance as a prognostic indicator for various chronic diseases in maintenance hemodialysis (MHD) patients [3–5]. Phase angle, a critical bioelectrical impedance indicator, significantly correlates with prognosis in various diseases. Although some studies indicate that phase angle has good correlation with the survival of chronic disease patients [6, 7], others have reported a correlation between phase angle and the risk for death in MHD patients. Here, we investigated the effect of phase angle level on predicting the risk factors for death in MHD patients.

## Methods and materials

### Methods study design and participants

**Fig. 1:**
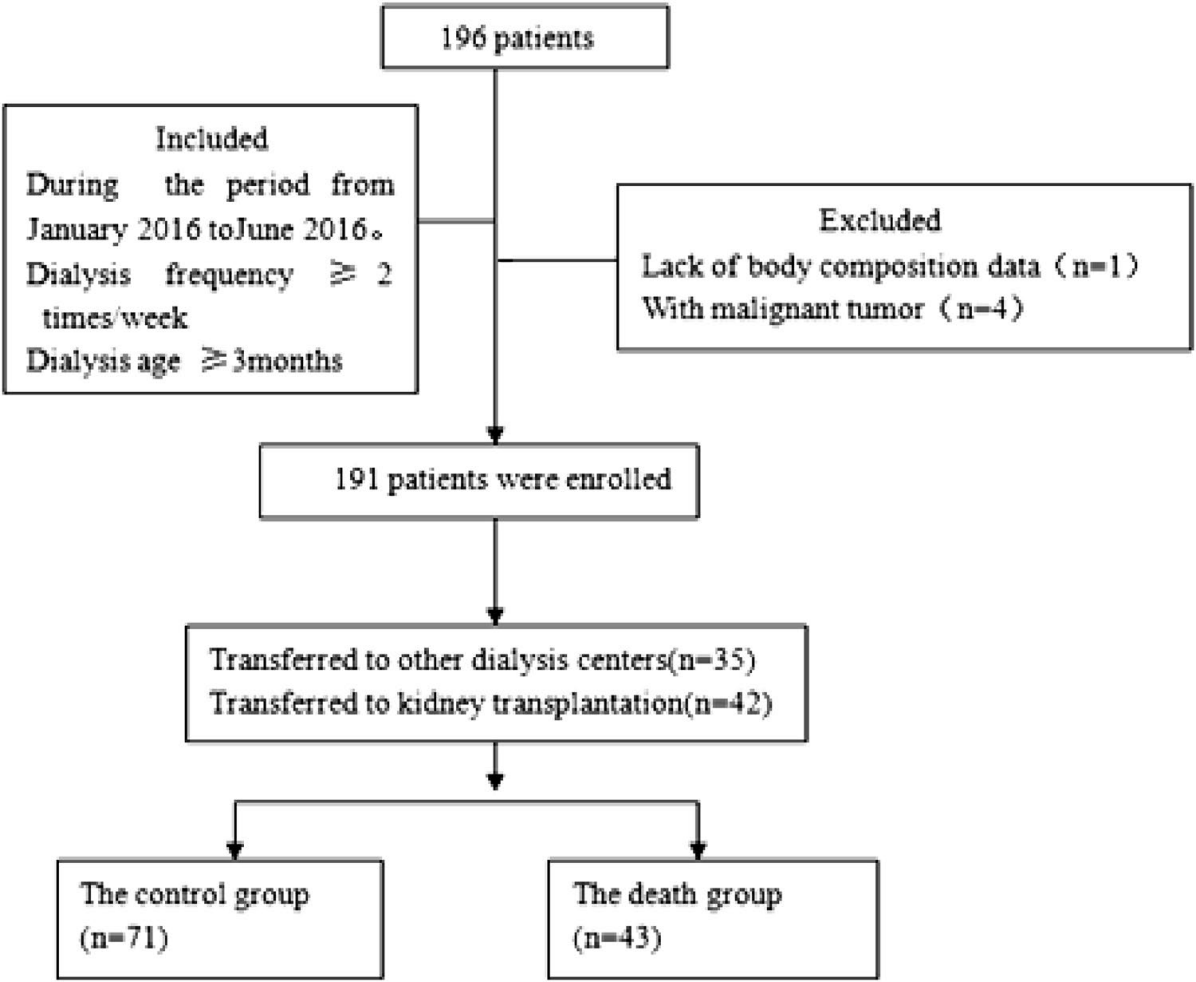
Flow chart of the selection of study participants

This study involved 196 patients who underwent regular hemodialysis at Shenzhen Traditional Chinese Medical Hospital, Guangdong Province, China, from January 2016 to June 2016. Follow-up data were collected from January 2016 to December 2021. Additional inclusion criteria were (1) at least 2 dialysis days/week and (2) a dialysis duration of ≥ 3 months. The exclusion criteria were (1) missing baseline or follow-up data and (2) cancer (Fig. 1).

### Data collection

#### Data collection and measures

General information, medical histories, and laboratory data were collected by physicians. Direct segmental multi-frequency bioelectrical impedance technology was used to analyze body composition (InBody S10, Shanghai, China) and to obtain the phase angle, fat-free mass (FFM), extracellular water/total body water (ECW/TBW), body mass index (BMI), and waist circumference (WC). The phase angle was obtained at a frequency of 50 Hz. The study adhered to the guidelines of the Declaration of Helsinki. The study was approved by the human research ethics committees of the involved hospitals. Investigators or persons authorized by the investigators explained the benefits and risks of trial participation to all study participants or their legal representatives or notaries. Trial data were stored in a safe in the office of the first author, who performed all statistical analyses.

#### Biochemical analysis

Serum samples were drawn at MHD commencement and analyzed locally. For all patients, serum creatinine (Cr), serum uric acid (UA), urea nitrogen (BUN), serum albumin (ALB), serum potassium (K), phosphorus (P), hemoglobin (HB), total cholesterol (TC), HDL-C, triglyceride (TG), and LDL-C were measured.

### Statistical analyses

Statistical analyses were performed using SPSS 22.0. Measurement data are presented as the mean ± SD. A *t* test was used to compare differences between groups with normally distributed data. The rank-sum test was used to compare differences between groups with non-normal data distributions. Differences between data groups expressed as proportions were compared using the *χ*^2^ test. Using a receiver operating characteristic curve (ROC) and area under the curve (AUC) analyses, cut-off values were calculated. Using the optimal ROC cut-off values, chi-square analysis was performed, and odds ratios and 95% confidence intervals (95% CIs) were calculated. Kaplan‒Meier and log-rank tests were used for univariate survival analysis. A Cox proportional risk regression model was used for multivariate analysis using the backwards elimination method to analyze risk factors in the variables. The test level was *ɑ* = 0.05. *P* < 0.05 indicated statistically significant differences.

## Results

Of the participants recruited into the study, 43 died. The most common causes of death in MHD patients were cardiovascular disease (67.44%, 29 cases) and stroke (18.6%, 8 cases). Other causes of death were infection (1 case), hyperkalemia (2 cases), systemic failure (2 cases), and gastrointestinal bleeding (1 case).

The age of the participants in the death group was significantly higher than that in the control group (*P* = 0.001). There were more diabetic patients in the death group than in the control group (*P* = 0.022). Compared with the control group, the death group had significantly different ECW/TBW (*P* = 0.000), phase angle (*P* = 0.000), ALB, Ca, and TG values. FFM, BMI, WC, protein, Cr, BUN, K, HB, P, UA, HDL-C, LDL-C, and TC did not differ significantly between the groups (*P* >  = 0.05, Table 1).

**Table 1:** Comparison of the two patient groups

Variables	The control group (*n* = 71)	The death group (*n* = 43)	*P* value
Sex			0.498
Male, *n* (%)	40 (56.3%)	27 (62.8%)	
Female, *n* (%)	31 (43.7%)	16 (37.2%)	
Age (years)	53.94 ± 14.33	63.56 ± 13.84	0.001
Dialysis vintage (months)			0.469
3–12 months	16 (22.5%)	14 (32.6%)	
13–36 months	21 (29.6%)	10 (23.3%)	
37–60 months	8 (11.3%)	7 (16.3%)	
> 60 months	26 (36.6%)	12 (27.9%)	
Diabetes			0.022
Yes	24 (33.8%)	23 (53.5%)	
No	47 (66.2%)	20 (46.5%)	
Dialysis frequency			0.718
Three times a week	70 (98.6%)	42 (97.7%)	
Twice a week	1 (1.4%)	1 (2.3%)	
KT/V	1.43 ± 0.42	1.23 ± 0.24	0.005
FFM (kg)	43.48 ± 8.19	45.34 ± 8.15	0.433
ECW/TBW	0.378 ± 0.019	0.397 ± 0.018	0.000
BMI (kg/m^2^)	22.50 ± 2.46	22.39 ± 3.66	0.905
Phase angle	5.90 ± 1.35	4.72 ± 1.85	0.000
WC (cm)	82.07 ± 11.46	81.93 ± 12.21	0.951
ALB (g/L)	40.21 ± 2.94	37.15 ± 7.01	0.003
LDL-C (mmol/L)	2.07 ± 0.74	2.45 ± 1.34	0.123
HDL-C (mmol/L)	1.04 ± 0.28	0.99 ± 0.49	0.607
Ca (mmol/L)	2.24 ± 0.29	2.09 ± 0.30	0.000
TG (mmol/L)	1.68 ± 0.95	1.21 ± 0.40	0.032
Cr (umol/L)	909.98 ± 430.07	866.16 ± 263.61	0.436
BUN (mmol/L)	23.50 ± 6.90	25.46 ± 8.17	0.219
UA (mmol/L)	363.70 ± 157.56	418.89 ± 124.93	0.079
P (mmol/L)	1.89 ± 0.68	2.13 ± 0.82	0.448
HB (g/L)	110.17 ± 15.00	107.48 ± 18.62	0.417
TC (mmol/L)	0.403 ± 0.012	0.404 ± 0.02	0.853
K (mmol/L)	4.89 ± 0.72	5.18 ± 1.18	0.121

Correlations of body composition and biochemical variables with phase angle were analyzed (Table 2). ECW/TBW (*R* =  − 0.923, *p* = 0.000) was negatively correlated with the phase angle. FFM (*R* = 0.258, *p* = 0.009) and ALB (*R* = 0.247, *p* = 0.012) were positively correlated with phase angle

**Table 2:** Correlation of body composition and biochemical variables with phase angle

Variables	*R*	*P* value
BMI	0.112	0.269
FFM	0.258	0.009
WC	− 0.005	0.959
ECW/TBW	− 0.923	0.000
ALB	0.247	0.012

**Fig. 2:**
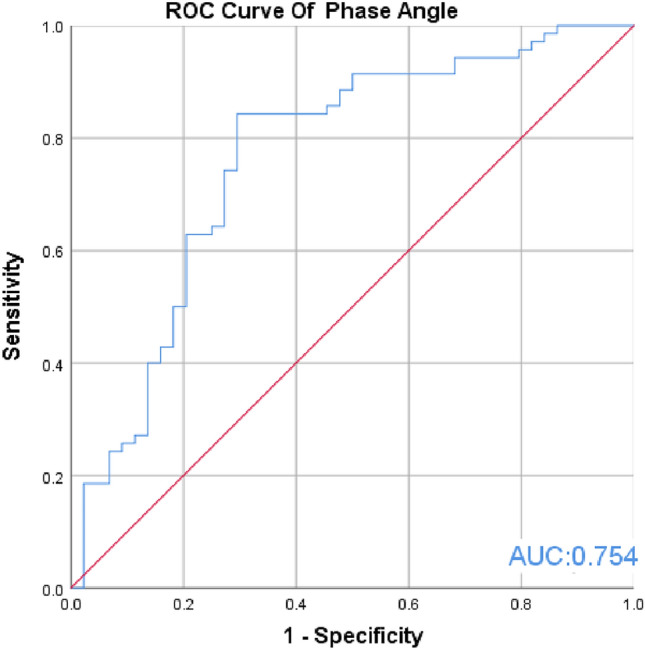
ROC curve for the ability of phase angle to predict death

The ROC analysis (Fig. 2) showed an AUC of 0.754 (95% CI = 0.151, 0.346), and the optimal cut-off value for predicting PA was determined to be 4.50°, with a sensitivity of 0.488 and specificity of 0.901.

**Fig. 3:**
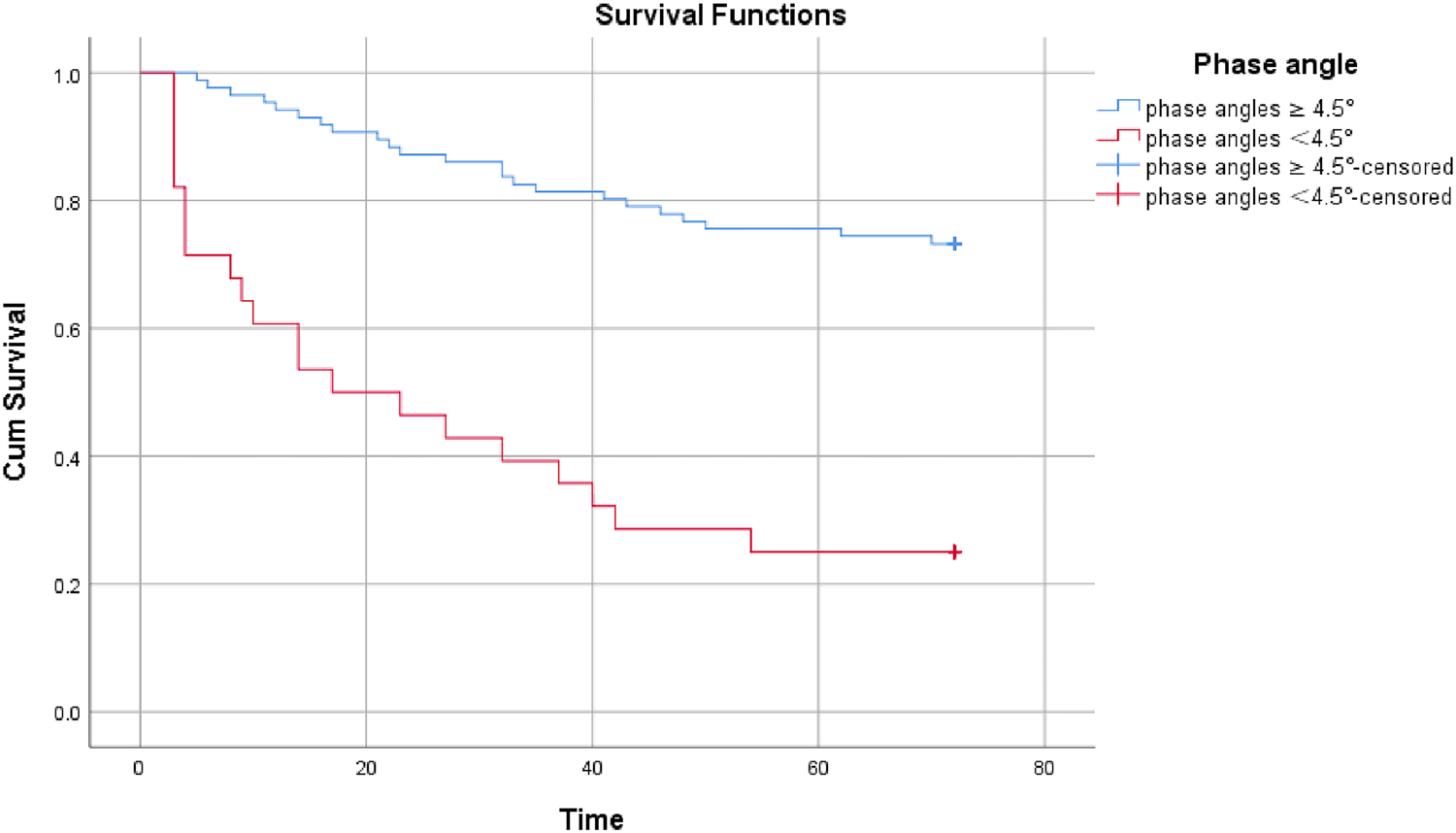
The Kaplan‒Meier curves based on phase angle

According to the phase angle cut-off value of 4.5°, the study population was classified into two groups for analysis. Kaplan‒Meier survival curves (Fig. 3) demonstrated a significant improvement in survival time (*p* = 0.000) for patients with phase angles ≥ 4.5° compared to those with phase angles < 4.5°. Specifically, the mean survival time for the phase angle ≥ 4.5° group (*n* = 86) was 60.581 months (95% CI = 56.148, 65.041), while that for the comparison group (*n* = 28, phase angle < 4.5°) was 30.643 months (95% CI = 20.512, 40.774). Additionally, similar results were observed for patients with old age (≥ 65 years, *p* = 0.008), diabetes (*p* = 0.0014), and hypoproteinemia (Alb < 35 g/L, *p* = 0.000).

All variables in the Cox regression model met the PH assumption; therefore, a Cox proportional hazards model was used, and the results are presented in Table 3. Of the six independent variables included, all but diabetes had significance as determined by *t* test (*P* values less than 0.05), and the significance of increased age was marginal (*p* = 0.059). These findings imply that phase angle, albumin level, dialysis vintage, KT/V, and age all have a significant impact on the survival time of dialysis patients. The risk of death was increased by 2.647 times for those with a low phase angle (< 4.5°) (HR = 2.647, 95% CI = 1.247, 5.618, *p* = 0.011) and by 6.188 times for those with hypoalbuminemia (< 35 g/L) (HR = 6.188, 95% CI = 2.611, 14.662, *p* = 0.000). KT/V was also found to be a risk factor for MHD patient mortality, with higher KT/V values leading to a lower risk of death and a 77% decrease in death risk for every additional unit of KT/V. Although the risk of dialysis-related death increased with age, there was a 2.6% decrease in this risk with each one-month increase in dialysis vintage.

**Table 3:** Independent predictors of survival: multivariate Cox regression

Variables	HR	*P* value	95% CI
Lower phase angle (< 4.5°)	2.647	0.011	1.247–5.618
Age	1.029	0.059	0.999–1.061
Hypoalbuminemia (< 35 g/L)	6.188	0.000	2.611–14.662
Dialysis vintage	0.974	0.000	0.962–0.986
Diabetes	0.562	0.144	0.259–1.217
KT/V	0.230	0.042	0.056–0.947

## Discussion

According to previous studies, phase angle has been identified as an independent risk factor associated with malnutrition in CKD patients [8]. In line with this, our study findings suggest that lower phase angle can be utilized as a predictive marker for mortality risk in the MHD population. Currently, phase angle is utilized in cancer, liver cirrhosis, hemodialysis, and surgical outcome studies, in which it has been found to have an association with poor prognosis [9]. As a noninvasive, affordable, objective, and quantifiable measurement that does not require radiation exposure, phase angle is a convenient parameter for assessing nutritional status. Phase angle reflects cell membrane integrity and overall cellular health by indicating cell resistivity and can serve as a sensitive indicator of malnutrition to detect clinical nutritional status changes [10, 11]. The European Society for Parenteral and Enteral Nutrition recognizes the prognostic value of phase angle in patients requiring nutritional therapy, highlighting that lower phase angles are associated with worse outcomes in ICU patients, while higher phase angles are associated with improved survival [10, 11]. Phase angle can reflect early cellular dysfunction, which can indicate the occurrence and development of diseases and disorders, such as inflammation or cancer, as well as prognosis. The phase angle is calculated using the following formula: arctangent (capacitive reactance/impedance × 180°/π). The theoretical basis underlying how phase angle reflects somatic health level is related to the “conductive part” of the human body, which is also reflected in other measures, such as ICW, ECW, and other conductive components, at the cellular level that produce resistance to current [12, 13]. Capacitance across the cell membrane produces capacitive reactance to current, leading to phase shifts that are out of step with the voltage, which is associated with the number, integrity, and continuity of cell membranes, ECW, and ICW. Therefore, the phase angle serves as a comprehensive reflection of the influence of inflammation, immunity, nutrition, and other factors on the health and function of cells. In a study conducted by Gonzalez et al., a reduction in phase angle due to changes in ECW was found in healthy subjects, indicating a potential correlation of low phase angles with malnutrition and chronic inflammation in dialysis patients [14]. Bioelectrical impedance analysis is a noninvasive, reliable technique utilized for estimating body composition and predicting various conditions, including type 2 diabetes and muscle dysfunction, as well as hydration and nutritional status [15]. Phase angle, as an indicator of bioelectrical impedance, significantly correlates with cancer prognosis. Moreover, a study reported that a decreased phase angle was associated with a greater risk of PEW and frailty in MHD patients [16]. A low phase angle often reflects reduced extracellular matrix and increased apoptosis, in addition to the amount of water in a patient's body, indicating poor cell function and membrane integrity [17]. The mechanism underlying why lower phase angle can serve as a predictor of the prognosis of MHD patients warrants further investigation.

Cardiovascular disease remains the leading cause of all-cause mortality in hemodialysis patients, likely due to their unique risk factors, such as rapid electrolyte changes, prolonged QT intervals, calcium and phosphorus metabolism disorders, sympathetic excitation, uremic toxin invasion, and hemodialysis-related hemodynamic changes [18, 19]. In this study, cardiovascular diseases, including heart failure, arrhythmia, ischemic heart disease, and sudden cardiac death, were the most common causes of death (67.44%) in MHD patients. Based on the KM analysis, diabetes significantly affected the survival rate of dialysis patients. However, in the Cox multivariate analysis, diabetes was not identified as an independent risk factor. This suggests that the impact of diabetes on the survival of dialysis patients is not significant when other potential risk factors are controlled for and may be caused by its interaction with other underlying risk factors. Additionally, dialysis vintage and old age were risk factors for death in MHD patients. This may be attributed to an increased likelihood of cardiovascular disease occurrence with increasing age, as well as potential nutritional losses linked to prolonged dialysis duration.

Serum albumin levels have been identified to directly reflect nutritional status, and protein-energy malnutrition has been found to be common in MHD patients, with prevalence rates ranging from 23 to 73%. Cox regression analysis also indicated that an albumin level of < 35 g/L was an independent risk factor for death in hemodialysis patients. Reduced albumin levels may result from inadequate intake of nutrients, uremic toxin invasion, delayed gastric emptying, nutrient loss during dialysis, endocrine disorders, metabolic acidosis, and increased energy consumption due to complications, which exacerbate malnutrition in MHD patients and increase mortality [20]. The International Association of Renal Nutrition and Metabolism defines protein-energy wasting (PEW) in hemodialysis patients, with one of the main diagnostic criteria being a serum biochemical index of ALB < 35 g/L. Among MHD patients, a decrease of 1 g/dL in serum albumin was associated with a significant 47% increase in mortality risk.

The adequate provision of dialysis is a critical factor in ensuring the long-term health of dialysis patients. The parameter KT/V serves as an indicator of the sufficiency of dialysis [21]. Insufficient dialysis can result in volume overload and electrolyte metabolism disorders, leading to various complications, such as hypertension, left ventricular hypertrophy, congestive heart failure, calcium and phosphorus metabolism disorders, and vascular calcification, which can ultimately contribute to mortality.

This research has certain limitations. First, our research was limited to only patients from a single hemodialysis center. The sample size was relatively small, yet the results are consistent with those of previous clinical research. In future research, more disease cases from different hemodialysis centers should be collected for study. Second, due to the cross-sectional nature in this study, correlation analysis was conducted using only age, dialysis vintage, hypoproteinemia, KT/V, diabetes status, phase angle, and patient survival as variables. Causal relationships between these variables could not be determined but could be further explored by future studies.

## Conclusion

Here, we found that phase angle as well as hypoproteinemia might predict survival in MHD patients. Further investigation involving a larger population and the factors studied here alone or in comparison with other prognostic factors is needed to confirm our findings and to assess their accuracy in MHD patients.

## Data Availability

The authors had full access to all data. The datasets used and/or analyzed during the current study are available from the corresponding author on reasonable request.

## Abbreviations

MHD: Maintenance hemodialysis
FFM: Fat-free mass
ECW/TBW: Extracellular water/total body water
ECW: Extracellular water
ICW: Intracellular water
WC: Waist circumference
CKD: Chronic kidney disease
ESRD: End-stage renal disease
RRT: Renal replacement therapy
BMI: Body mass index
Cr: Creatinine
UA: Uric acid
BUN: Urea nitrogen
ALB: Albumin
K: Potassium
P: Phosphorus
HB: Hemoglobin
TC: Total cholesterol
TG: Triglyceride
ROC: Receiver operating characteristic
AUC: Area under the curve
PEW: Protein-energy wasting
